# Effects of *Bacillus coagulans* on Growth Performance, Digestive Enzyme Activity, and Intestinal Microbiota of the Juvenile Fourfinger Threadfin (*Eleutheronema tetradactylum*)

**DOI:** 10.3390/ani15040515

**Published:** 2025-02-11

**Authors:** Anna Zheng, Jiaqin Hu, Evodia Moses Mkulo, Minxuan Jin, Linjuan Wang, Huijuan Zhang, Baogui Tang, Hui Zhou, Bei Wang, Jiansheng Huang, Zhongliang Wang

**Affiliations:** 1 College of Fisheries, Guangdong Ocean University, Zhanjiang 524088, China; 2 Guangdong Provincial Key Laboratory of Aquatic Animal Disease Control and Healthy Culture, Zhanjiang 524088, China; 3 Agro-Tech Extension Center of Guangdong Province, Guangzhou 510520, China

**Keywords:** *Eleutheronema tetradactylum*, *Bacillus coagulans*, growth performance, intestinal microbiota, probiotics

## Abstract

The effects of *Bacillus coagulans* (BC) T-21 on growth performance, intestinal digestive enzyme activity, intestinal morphology, and intestinal microbiota of juvenile fourfinger threadfin (*Eleutheronema tetradactylum*) were investigated in the present study. Healthy juvenile *E. tetradactylum* with an initial body weight of 4.2 ± 0.5 g were fed a basal diet sprayed with 1 × 10^8^ cfu/g *B. coagulans* for eight weeks, and the growth parameters, intestinal digestive enzyme activities, HE-stained intestinal sections, and intestinal microbiota of the juvenile fish were measured. The differences in the feed conversion ratio between the experimental and control groups (fed the basal diet) were extremely significant (*p* < 0.01), whereas the differences in weight gain rate, specific growth rate, survival rate, and condition factor were significant (*p* < 0.05). In the experimental group, trypsin and amylase activities decreased significantly, whereas there were no significant differences in lipase activity between the two groups. Compared to the control group, the height of the intestinal villi was greater. No significant differences were observed in the diversity of intestinal microbiota and microbial species at the genus level (*p* > 0.05). Based on the function prediction analysis, the count values for the glycan biosynthesis, metabolism, and digestive system pathways were significantly increased in the experimental group (*p* < 0.05). However, there were no significant differences in the counts of other functional pathways (*p* > 0.05). These results indicate that dietary *B. coagulans* supplementation is effective in promoting the growth performance and intestinal health of juvenile *E. tetradactylum*.

## Simple Summary

This study examines the effects of adding Bacillus coagulans (a type of beneficial bacteria) to the diet of juvenile fourfinger threadfin fish (*Eleutheronema tetradactylum*) to improve their growth and gut health. Over an eight-week period, the fish were fed a diet treated with this bacterium, and their growth, digestive enzyme activity, gut structure, and gut bacteria were measured. Results showed that the fish in the experimental group grew better and had a more efficient feed conversion compared to the control group. However, the activities of certain digestive enzymes decreased, and there were no significant changes in the diversity of gut bacteria. The gut structure improved, with the fish having taller intestinal villi. Additionally, certain biological pathways linked to digestion and metabolism were more active in the experimental group. Overall, supplementing the fish’s diet with *B. coagulans* led to better growth and gut health, which could be valuable for improving fish farming practices and promoting healthier aquatic species.

## 1. Introduction

*Eleutheronema tetradactylum*, commonly known as the fourfinger threadfin, belongs to Polynemidae, *Eleutheronema* and is mainly distributed in the Indo-West Pacific, where it ranges from the Persian Gulf to Papua New Guinea and northern Australia [1]. The fourfinger threadfin exhibits broad adaptability to varying salinity levels, enabling its cultivation in both seawater and brackish water. It demonstrates rapid growth, and its flesh is highly regarded for its freshness, tenderness, and exceptional nutritional value. As a result, the species is highly valued by both consumers and aquaculture producers for its substantial economic potential. Recognized as a key aquaculture species by the Food and Agriculture Organization, large-scale seedling production of fourfinger threadfin has been successfully implemented in Guangdong, China [2].

Currently, the fourfinger threadfin is primarily cultivated in pond systems. However, their cultivation is frequently associated with challenges such as the dissolution of uneaten feed, the accumulation of fish excreta, and overfeeding [3]. These issues contribute to the eutrophication of the aquatic breeding environment, leading to a significant increase in metabolite concentrations and organic matter. The widespread use of antibiotics and disinfectants to address recurrent disease outbreaks has raised concerns regarding aquatic safety. In response to these challenges, probiotics have increasingly emerged as the primary approach for the prevention and control of aquatic diseases, supplanting traditional antibiotic treatments.

*Bacillus coagulans*, a spore-forming, gram-positive bacterium, is commonly used as a feed additive in aquaculture due to its unique combination of lactic acid bacteria and Bacillus properties [4,5]. It has demonstrated positive effects on immune function, disease resistance, and growth across a range of aquatic organisms, including common carp (*Cyprinus carpio*) [5], grass carp (*Ctenopharyngodon idella*) [6], Nile tilapia (*Oreochromis niloticus*) [7], grouper (*Epinephelus coioides*) [8], and shrimp (*Litopenaeus vannamei*) [9]. *B. coagulans* supports the regulation of microbiota, enhances immune function, and promotes growth by colonizing the intestines, thereby improving intestinal health and providing resistance to infection. Additionally, *B. coagulans* has been found to play a significant role in mitigating stress and immune damage induced by heavy metal ions, such as copper and cadmium [10,11,12,13].

In this study, *B. coagulans* was sprayed into the commercial feed in order to compare and analyze the physiological condition of juvenile *E. tetradactylum* before and after supplementation. The primary objective was to assess the effects of *B. coagulans* on growth performance, intestinal health, and the structure of the microbial community in juvenile *E. tetradactylum*. The findings aim to contribute scientific insights toward enhancing aquaculture breeding technologies and guiding the development of novel feed additives.

## 2. Materials and Methods

### 2.1. Feed Ingredients and Formulation

*B. coagulans* T-21 strain with a concentration of 5 × 10^9^ CFU per gram was supplied by Kunming Aikete Biotechnology Co., Ltd. (Kunming, China). The juvenile *E. tetradactylum* (mean initial body weight 4.2 ± 0.5 g) was purchased from a commercial fish farm (Zhanjiang, China). Basal feed was purchased from Weifang Santong Biological Engineering Co., Ltd. (Weifang, China). The main nutrients in the basal feed were crude protein content ≥ 55%, crude fat content ≥ 8%, crude fiber content ≤ 3%, and crude ash content ≤ 16%).

### 2.2. Experimental Design and Feeding Trial

Prior to the trial, 420 healthy *E. tetradactylum* were randomly selected and kept in 500 L aerated tanks for a period of 1 week for acclimation. After acclimation, juvenile fish were randomly divided into 2 treatment groups, with 3 replicates in each group and 70 juveniles in each replicate.

*B. coagulans* T-21 was incubated with shaking in MRS broth at 42 °C for 48 h. The cultures were then centrifuged at 5000× *g* for 10 min. The bacterial pellet was washed three times with sterile phosphate-buffered saline and resuspended to a final concentration of 1 × 10^9^ CFU/mL. The bacterial suspension was diluted and evenly sprayed onto the basal feed at a concentration of 1 × 10^8^ CFU/g. The feed was then dehydrated and stored at −20 °C until use [14]. The fish were fed three times a day (8:00, 12:00, and 17:00) for an 8 week feeding trial. During the experiment, water temperature (27–29.5 °C), dissolved oxygen (DO > 6 mg/L), pH (7.8–8.0), ammonia nitrogen (less than 0.3 mg/L), and salinity (27–30 ppt) were monitored daily using a water quality analyzer (Zhanjiang, China).

### 2.3. Sample Collection

At the end of the feeding trial, the fish selected randomly from each tank were sampled and weighed after fasting for 24 h. The whole intestine of sampled fish anesthetized with eugenol (50 mg/L) was dissected under sterile conditions, and the extraintestinal adipose tissue was stripped. The whole intestine was used to determine the intestinal digestive enzyme activity and intestinal microbiota. The intestines were fixed in 4% paraformaldehyde to measure intestinal morphology.

### 2.4. Growth Performance

Samples of 10 juveniles selected randomly from each tank were weighed at the end of the experiment, and the specific growth rate (SGR), weight gain rate (WGR), survival rate (SR), feed conversion ratio (FCR), visceral body index (VSI), and condition factor (CF) were calculated using the following formulae:

SGR(%) = (Ln final weight − Ln initial weight)/feeding days × 100

WGR(%) = (final weight − initial weight)/initial weight × 100

SR(%) = final/initial × 100

FCR(%) = total weight of feed consumed/total weight gain

VSI(%) = visceral weight/fish weight × 100

CF(%) = final body weight (g)/final body length (cm)^3^ × 100

### 2.5. Intestinal Morphology

The fixed intestinal tissues were soaked in 4% paraformaldehyde, paraffin-embedded and sectioned (5–7 μm), deparaffinized by xylene, stained with hematoxylin and eosin (H&E), air-dried, and then covered and observed with an optical microscope (Nikon 80i) at Wuhan Saiweier Biotechnology Co., Ltd. (Wuhan, China). Intestinal villi height was measured using ImageJ (win64) to assess the effects of *B. coagulans* on the intestinal structure by evaluating villi length. Nine intestinal samples were randomly selected from each group, and three measurements of villi height were taken from each sample.

### 2.6. Digestive Enzyme Activity

The intestinal digestive enzyme activities (trypsin Try, amylase AMS, lipase LPS) of juvenile *E. tetradactylum* were assayed by different test kits as listed in the following [15].

Trypsin-specific activity was assayed by using a trypsin kit (Nanjing Jiancheng Bioengineering Institute, No. A080-2) (Nanjing, China). The activity unit is defined as the amount of trypsin in 1 mg of protein that causes a 0.003 increase in absorbance within 1 min at 37 °C and pH 8.0.

Amylase-specific activity was assayed by using an amylase kit (Nanjing Jiancheng Bioengineering Institute, No. C016-1) (Nanjing, China). The activity unit is defined as the amount of amylase in 1 mg of protein that hydrolyzes 10 mg of starch substrate in 30 min at 37 °C.

Lipase-specific activity was assayed by using a lipase kit (Nanjing Jiancheng Bioengineering Institute, No. A054-1) (Nanjing, China). The activity unit is defined as the amount of lipase in 1 mg of protein that hydrolyzes 1 μmol of triglyceride substrate at 37 °C.

### 2.7. Intestinal Microbiota Analysis

Intestinal microbial DNA was extracted using the QIAamp PowerFecal DNA Kit (Guangzhou GeneDenovo Technology Co., Ltd., Guangzhou, China), following the manufacturer’s instructions. The DNA was then amplified from the V3-V4 hypervariable region of the 16S rRNA gene using the universal primers 338F (ACTCCTACGGGAGGCAGCA) and 806R (GGACTACHVGGGTWTCTAAT) for library construction. Sequencing was conducted on an Illumina MiSeq™ PE 300 system with high throughput at Guangzhou GeneDenovo Technology Co., Ltd. (Guangzhou, China).

To obtain clean, high-quality reads, the raw reads were quality-filtered. Additionally, for operational taxonomic units (OTUs), a sequence similarity threshold of 97% was applied. Sequencing data analysis included α-diversity assessment using the Shannon and Simpson indices, β-diversity using principal coordinate analysis (PCoA) and non-metric multidimensional scaling (NMDS), and analysis of microbial differences among groups at the phylum and genus. The Phylogenetic Investigation of Communities by Reconstruction of Unobserved States (PICRUSt2) was used to predict the Kyoto Encyclopedia of Genes and Genomes (KEGG) functions for the sequencing data, and the abundance of each functional category was subsequently calculated.

### 2.8. Statistical Analysis

The data were analyzed by an independent sample T-test using SPSS 24.0 and expressed as “mean ± standard deviation”. *p* < 0.05 indicated that the difference was significant.

## 3. Results

### 3.1. Growth Performance

As shown in Table 1, the fish fed with *B. coagulans*-supplemented diets showed significant improvements in growth performance. Specific growth, weight gain, survival, and condition factors were significantly higher in the experimental group than those in the control group (*p * <  0.05). The feed conversion ratio (FCR) was significantly lower in the experimental group (1.29 ± 0.02) than the control group (1.57 ± 0.02), indicating that the fish fed with *B. coagulans* had a more efficient conversion of feed into body mass (*p* < 0.01). However, no significant differences were observed in the visceral body index (VSI), with values of 5.76 ± 0.26 for the experimental group and 4.96 ± 0.57 for the control group.

### 3.2. Intestinal Histology

**Figure 1:**
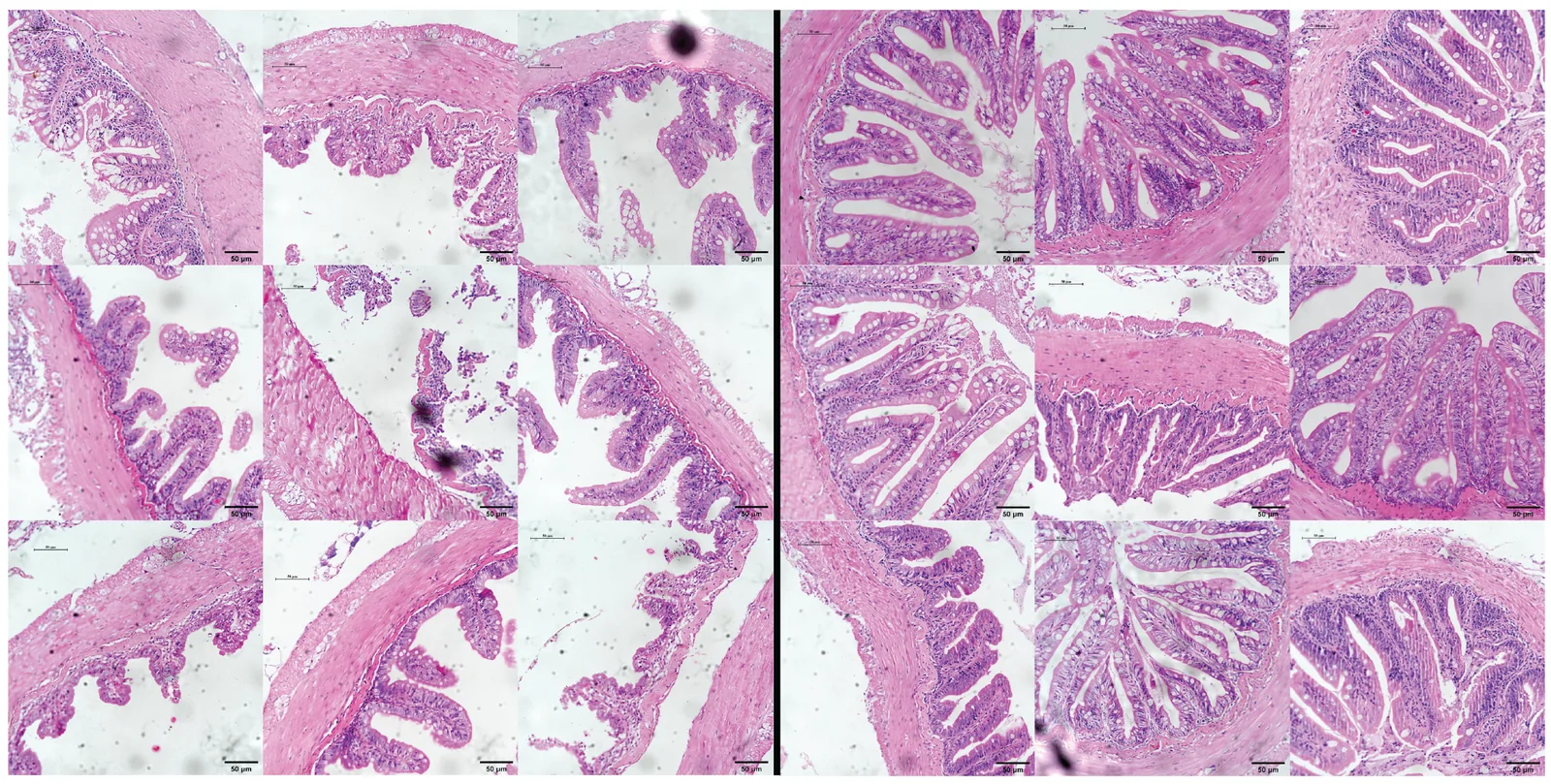
Intestinal morphology of *E. tetradactylum* fed diets supplemented with *B. coagulans*. The left images represent the control group, and the right images represent the experimental group. Scale bars represent 50 μm.

Light microscopy was used to assess the morphological features of the intestine in juvenile *E. tetradactylum*. Histological examination of the intestinal villi revealed that the villus height in the *B. coagulans* group was significantly greater, and the villi appeared more uniform compared to those in the control group (Tables S1 and S2; Figure 1).

### 3.3. Digestive Enzyme Activity

Table 2 illustrates the effect of dietary supplementation with *B. coagulans* on digestive enzyme activity in the intestine. Compared to the control group, the experimental group exhibited a significant decrease in trypsin activity (*p * <  0.05) and a highly significant decrease in amylase activity (*p * <  0.01). However, no significant difference in lipase activity was observed between the two groups.

### 3.4. Intestinal Microbiota Analysis

#### 3.4.1. High-Throughput Sequencing

**Figure 2:**
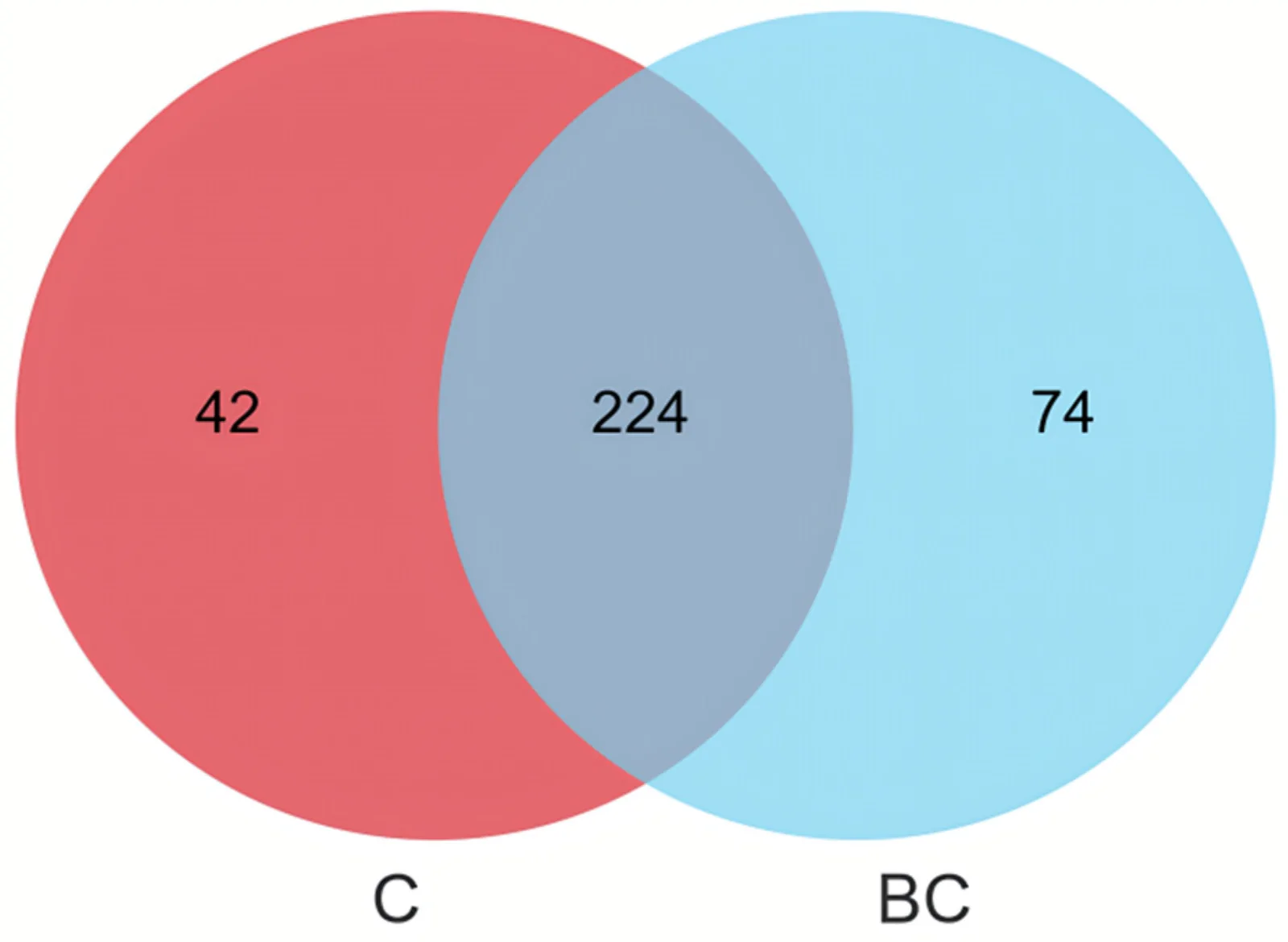
Effects of intestinal microbiota of *E. tetradactylum* supplemented with *B. coagulans* diets, Venn diagram of intestinal microbial OTUs.

We used 16S high-throughput sequencing to analyze changes in the intestinal microbiota of *E. tetradactylum* after 8 weeks of continuous feeding with *B. coagulans*. The number of effective tags for subsequent analysis was 89,982 after splicing and filtering, with an average effective data rate of 95.23%. Among these OTUs, 224 were shared in the intestinal samples of *E. tetradactylum*, while 42 and 74 OTUs were unique to the control and experimental groups, respectively (Figure 2).

#### 3.4.2. Intestinal Microbiota Diversity Indices

The Shannon and Simpson indices in the *B. coagulans* dietary groups were lower than those in the control groups; however, this difference was not statistically significant (*p* > 0.05). Similarly, the Chao1 and ACE indices were higher in the *B. coagulans* dietary groups compared to the control groups, but the differences were not significant (*p* = 0.073 > 0.05) (Table 3).

**Figure 3:**
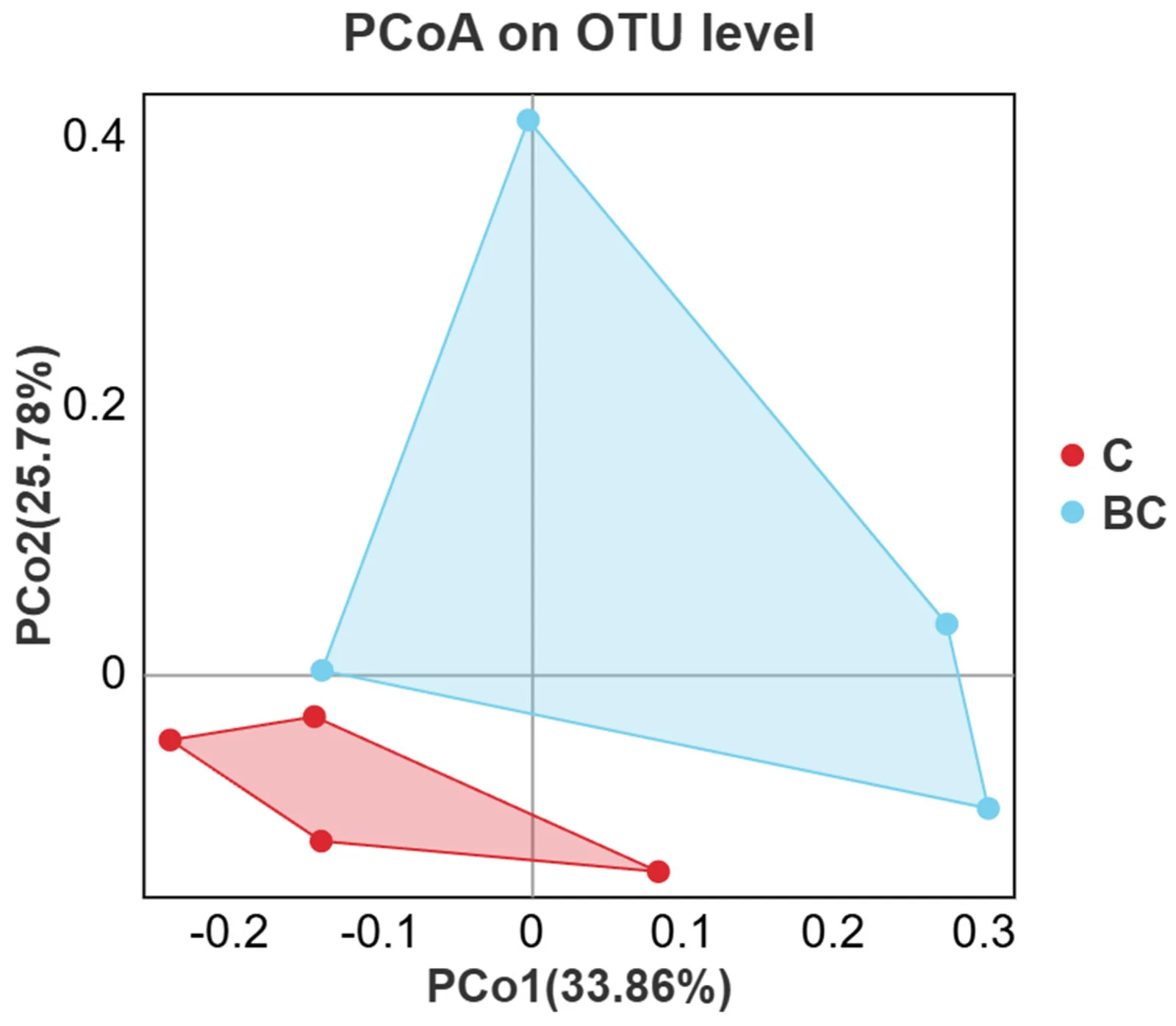
PCoA plot based on weighted UniFrac distance (C: the control group, BC: the experimental group).

The principal coordinate analysis (PCoA) based on weighted UniFrac distance showed that the *x*-axis (the first principal component contribution value) and *y*-axis (the second principal component contribution value) contributed 59.64% of the explanation. Clustering based on the weighted UniFrac distance of intestinal bacterial communities in *B. coagulans* dietary groups showed separation compared with the control group (Figure 3). Thus, *B. coagulans* caused no significant difference in the intestinal bacterial communities compared with the control group (*p*  = 0.081 >  0.05).

#### 3.4.3. Phylum and Genus Performance

**Figure 4:**
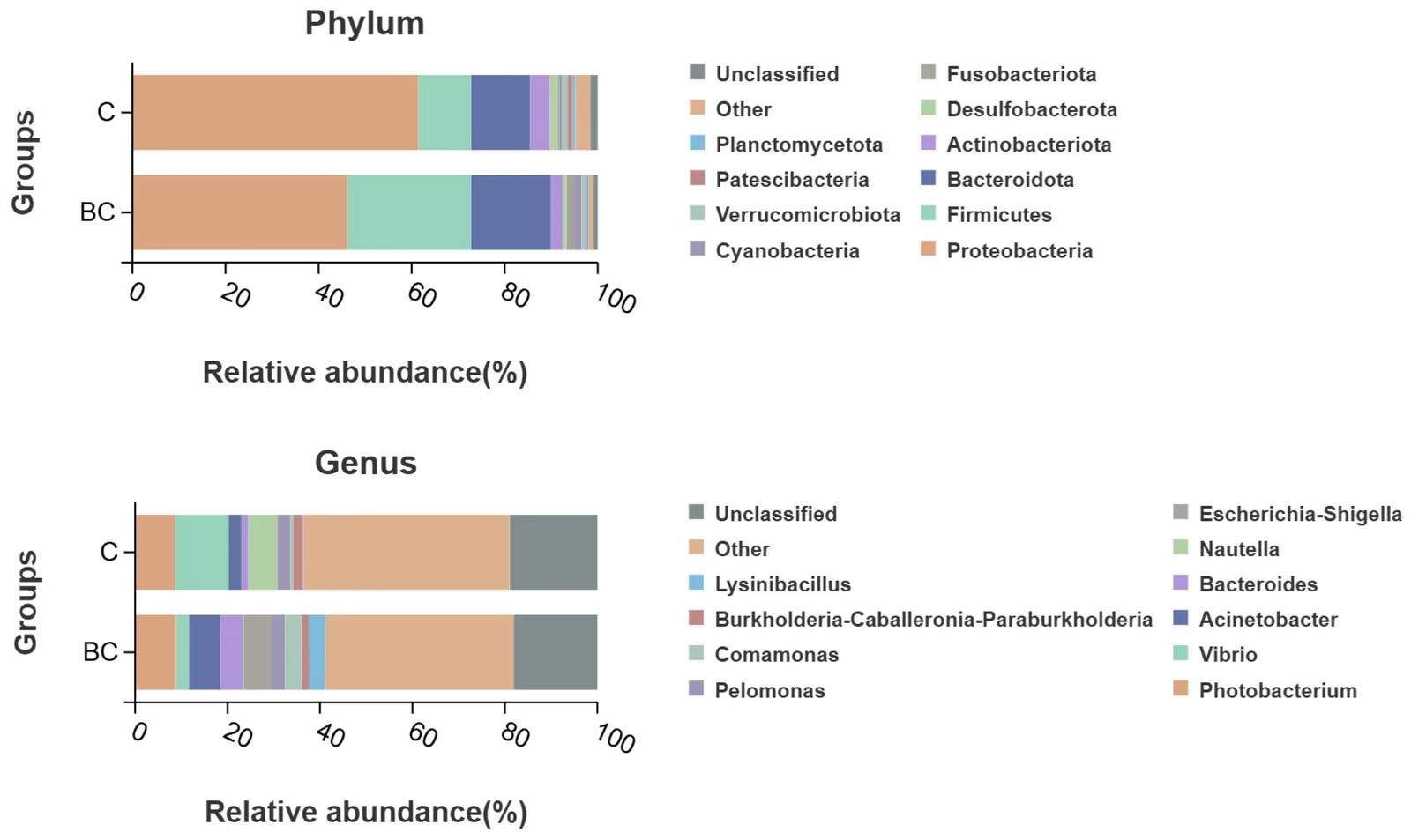
The relative abundance of intestinal bacteria of *E. tetradactylum* at the phylum and genus level.

At the phylum level, the dominant phyla across all experimental groups were Proteobacteria, Firmicutes, Bacteroidetes, Actinobacteria, Desulfobacteriota, Acidobacteria, Cyanobacteria, Verrucomicrobia, Patescibacteria, and Planctomycetota (Figure 4). Independent sample *t*-test analysis revealed a significant difference in the relative abundance of *Firmicutes* between the control and experimental groups (*p * <  0.05). At the genus level, the dominant genera in the control and experimental groups were *Vibrio*, *Photobacterium*, *Acinetobacter*, *Bacteroides*, *Nautella*, *Burkholderia-Caballeronia-Paraburkholderia*, *Escherichia-Shigella*, *Pelomonas*, *Comamonas*, and *Lysinibacillus* (Figure 4). There was no significant difference at the other genus levels (*p * = 0.087 >  0.05).

#### 3.4.4. Functional Prediction of Intestinal Microbiota

**Figure 5:**
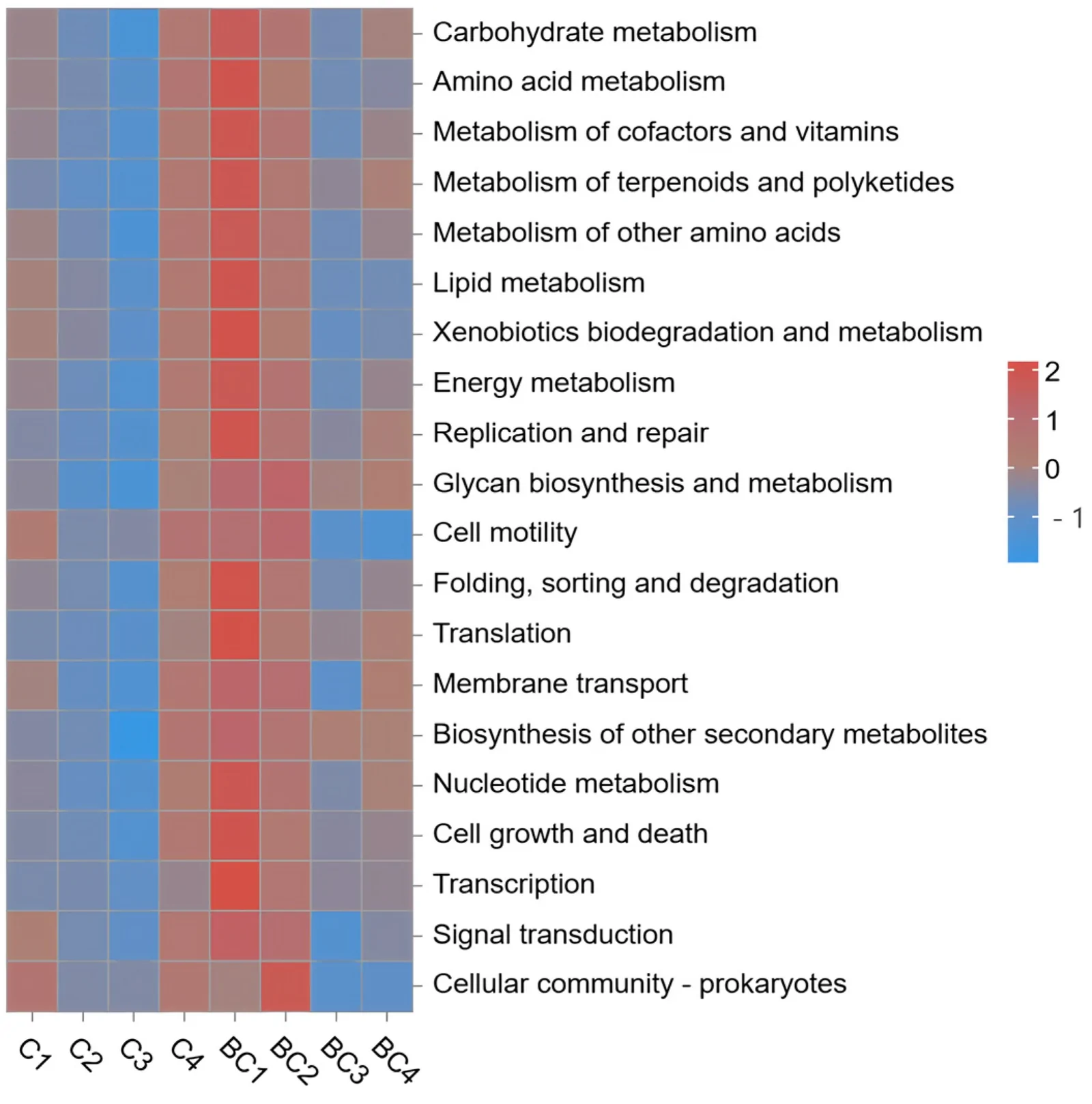
Functional prediction of intestinal microbiota between control and *B. coagulans* addition groups based on PICRUSt2.

The primary functional pathways of the intestinal microbiota of *E. tetradactylum*, ranked by abundance, included metabolism, genetic information processing, cellular processes, environmental information processing, organismal systems, and human diseases (Figure 5). Within the ‘metabolism’ category, the top three secondary pathways by abundance were carbohydrate metabolism, amino acid metabolism, and the metabolism of coenzyme factors and vitamins. The count values for ‘glycan biosynthesis and metabolism’ and ‘digestive system’ pathways were significantly increased in the experimental group (*p * <  0.05). Additionally, ‘signal molecules and interactions’ were introduced in the experimental group. No significant differences were observed in the count values of other functional pathways (*p* = 0.173 >  0.05).

## 4. Discussions

*B. coagulans* is a highly promising probiotic candidate among the various probiotics used as protein feed additives in the aquaculture industry, owing to its nonpathogenic and nontoxic characteristics [16]. Recent research has increasingly focused on the beneficial effects of probiotics on immunological activity, disease resistance, growth, and intestinal microbiota in various aquatic animals. However, there are no studies to date that specifically investigate the application of *B. coagulans* in *E. tetradactylum*.

This study assessed the effects of *B. coagulans* supplementation on the growth performance, intestinal health, digestive enzyme activity, and intestinal microbiota of *E. tetradactylum*. Previous evidence suggests that supplementing animal diets with Bacillus improves growth performance. For example, the inclusion of *B. licheniformis* in the diet of *Haliotis discus hannai* was found to significantly enhance growth compared to an unsupplemented control group [17]. Moreover, diets supplemented with *B. coagulans* have led to the effective growth performance of common carp (*Cyprinus carpio* L.) and Pacific white shrimp (*Litopenaeus vannamei*) [9,14]. Additionally, dietary supplementation with *B. coagulans* has shown similar improvements in growth performance in other animals, such as broiler chickens and piglets. Collectively, these findings underscore the beneficial role of probiotics in enhancing growth performance and overall health in various aquaculture species, including *E. tetradactylum*, highlighting their potential as valuable additives in aquaculture nutrition.

Fish digestion and nutrient absorption are significantly influenced by the morphology and functionality of the intestines [18]. Several morphological characteristics of the gastrointestinal tract, such as villi height and muscle structure, along with the activity of digestive enzymes like protease, amylase, and lipase, serve as indicators of gut health in fish [19,20]. It is also believed that an increase in the height or width of microvilli on the intestinal epithelial cells enhances the surface area available for nutrient absorption [21]. In this study, the intestinal villi of *E. tetradactylum* in the experimental group were taller and more uniform compared to those in the control group (Tables S1 and S2). This morphological improvement likely contributes to a larger nutrient absorption area, thereby facilitating more efficient nutrient digestion. Similarly, dietary supplementation of single- and multi-strain *Bacillus*-based probiotics improved all intestinal histomorphometric parameters (intestinal villi length, intestinal villi width, inter-villus space, and goblet cell number) in *Oreochromis niloticus* fingerlings [22].

One of the positive effects of feeding probiotics, prebiotics, and synbiotics to aquatic animals is the upregulation of digestive enzyme concentrations [23]. These feeding strategies can stimulate the production of digestive enzymes, leading to enhanced digestion, improved feed digestibility, and more efficient nutrient utilization. For example, the administration of probiotics, prebiotics, and synbiotics has been shown to boost digestive enzyme activities (such as protease, lipase, and amylase) in several cultured fish species [24,25,26]. Previous studies have also reported that dietary supplementation with *Bacillus* strains enhances the growth performance of species such as black rockfish (*Sebastes schlegelii*), triangular bream (*Megalobrama terminalis*), and Nile tilapia (*Oreochromis niloticus*) by increasing intestinal digestive enzyme activity, boosting liver antioxidant enzyme levels, and improving gut morphology [7,27,28]. In the current study, however, the activities of trypsin, amylase, and lipase in the *B. coagulans*-supplemented group were lower than those observed in the control group, exhibiting varying degrees of reduction. This discrepancy could be due to several factors, including differences in the probiotic strains used, variations in dosage and supplementation duration, or the specific physiological responses of the fish species examined. These factors may influence the efficacy of probiotic supplementation on digestive enzyme activity and overall gut health. Additionally, it is possible that the observed decrease in enzyme activity does not indicate reduced digestive efficiency. *B. coagulans* may have enhanced digestive performance through other mechanisms, such as modulation of gut microbiota, optimization of gut health, or improved nutrient absorption, which could compensate for the reduction in enzyme activity.

A growing body of evidence suggests that probiotics as feed additives can enhance the α-diversity of intestinal microbiota in various aquatic animals. It has been observed that during probiotic administration, the α-diversity of intestinal microbiota initially decreases before showing an increase. Similarly, long-term probiotic intervention appears to facilitate the recovery of beneficial microbiota that may be suppressed in earlier stages [29]. For instance, the introduction of *L. plantarum* and *B. subtilis* into water has been shown to promote the growth and boost nonspecific immunity of largemouth bass (*Micropterus salmoides*). This is achieved by stabilizing the gut microbiota, improving digestive and absorptive efficiency, and boosting anti-inflammatory and immune responses through the secondary metabolites produced by intestinal microbes [30]. The Shannon and Simpson indices, which are based on species richness and evenness, reveal that higher Shannon values are associated with lower Simpson values, indicating greater bacterial community diversity [31,32]. While *B. coagulans* may influence the growth of certain dominant phyla, leading to a simplified composition of the intestinal microbiota, the overall effect was not obvious, and no significant structural changes in the gut microbiota were observed. Chao and Ace are used to describe the estimators of community richness, and the larger the Chao1 and ACE indices, the higher the community richness [33]. The Chao1 and ACE indices showed a slight upward trend, indicating that *B. coagulans* might have a moderate effect in promoting the growth of intestinal microbial communities. Additionally, the principal coordinates analysis (PCoA) conducted in this study effectively revealed the main differences between samples. PCoA clustering further revealed that the intestinal microbiota in the control group exhibited high stability and uniformity, with greater similarity among individual samples. In contrast, the experimental group showed higher diversity, reduced stability, and possibly the emergence of new microbial populations.

Firmicutes are widely distributed in the intestine and are closely related to nutrient absorption and energy metabolism [34,35]. In the experimental group, the species richness of Firmicutes increased significantly compared to the control group, suggesting that *B. coagulans* may influence nutrient absorption and energy metabolism in fish by modulating the abundance of Firmicutes. Functional prediction analysis revealed significant improvements in ‘glycan biosynthesis and metabolism’ as well as the ‘digestive system’ in the experimental group. Glycans are critical components of the intestinal mucosal barrier, suggesting that *B. coagulans* enhances the degradation and utilization of intestinal glycans, thereby improving overall digestive function. Additionally, the observed increase in ‘signal molecules and interactions’ may facilitate communication between intestinal cells, further enhancing intestinal function and contributing to the improved growth performance seen in the experimental group. In conclusion, although *B. coagulans* did not significantly impact the species richness or evenness of the intestinal microbiota in *E. tetradactylum*, it appears to promote digestive efficiency and nutrient absorption within the intestine.

## 5. Conclusions

The findings of this study demonstrate that the inclusion of *B. coagulans* in the diet significantly enhances the growth of *E. tetradactylum* while reducing feed conversion efficiency. Despite the observed reduction in amylase and trypsin activities in the experimental group, the overall function of the digestive system improved. This can be attributed to the introduction of new signaling molecules and interaction pathways, which contributed to the coordination and stability of the intestinal microbiota. Additionally, the increased abundance of Firmicutes likely facilitated enhanced nutrient absorption and energy metabolism. Overall, *B. coagulans* proved effective in promoting the growth of juvenile *E. tetradactylum* while also improving intestinal morphology and the health of the gut microbiota.

## Figures and Tables

**Table 1:** Differences in growth performance indices of juvenile *E*. *tetradactylum*.

Parameter	Control	Experimental Group
SGR	3.71 ± 0.08	4.05 ± 0.04 *
WGR	190.05 ± 6.53	229.59 ± 4.91 *
SR	90.48 ± 2.22	95.24 ± 1.59 *
FCR	1.57 ± 0.02	1.29 ± 0.02 **
VSI	4.96 ± 0.57	5.76 ± 0.26
CF	3.56 ± 0.12	4.05 ± 0.06 *

Notes: SGR: specific growth rate; WGR: weight gain rate; SR: survival rate; FCR: feed conversion ratio; VSI: viscera-somatic index; CF: condition factor; data are expressed as the mean ± SD of three independent replicates, with each replicate consisting of measurements from 10 randomly selected fish per group. Values in the same row with different asterisk represent a significant difference (* *p * <  0.05/** *p * <  0.01), and the absence of letters indicates no significant difference between treatments.

**Table 2:** Effects of *B. coagulans* on intestinal digestive enzyme activities of juvenile *E. tetradactylum* (u/mg).

Parameter	Control	Experimental Group
Trypsin	37,354.71	6330.01 *
Amylase	6.85	4.3 **
Lipase	12.8	9.92

Data are expressed as the mean ± SD of three independent replicates, with each replicate consisting of measurements from 10 randomly selected fish per group. Values in the same row with different asterisk represent a significant difference (* *p * <  0.05/** *p * <  0.01), and the absence of letters indicates no significant difference between treatments.

**Table 3:** Alpha indices statistics.

Index	Control	Experimental Group
Reads	77,694	102,272
OUT	42	74
Shannon	7.16 ± 0.35	6.79 ± 0.78
Simpson	0.98 ± 0.009	0.97 ± 0.25
Chao1	1328.88 ± 97.48	1349.08 ± 118.09
Ace	1395.88 ± 83.71	1417.77 ± 118.06
Coverage	0.995 ± 0.001	0.996 ± 0.002

## Data Availability

The original contributions presented in this study are included in the article. Further inquiries can be directed to the corresponding author.

## Supplementary Materials

The following supporting information can be downloaded at: https://www.mdpi.com/article/10.3390/ani15040515/s1. Table S1: Individual intestinal villus height measurements and fish-level means in the control and *B. coagulans* groups; Table S2: Statistical analysis output for fish-level villus height comparison (IBM SPSS Statistics 24).
