# Correction: Zheng et al. Effects of *Bacillus coagulans* on Growth Performance, Digestive Enzyme Activity, and Intestinal Microbiota of the Juvenile Fourfinger Threadfin (*Eleutheronema tetradactylum*). *Animals* 2025, *15*, 515

**DOI:** 10.3390/ani16213402

**Published:** 2026-10-28

**Authors:** Anna Zheng, Jiaqin Hu, Evodia Moses Mkulo, Minxuan Jin, Linjuan Wang, Huijuan Zhang, Baogui Tang, Hui Zhou, Bei Wang, Jiansheng Huang, Zhongliang Wang

**Affiliations:** 1 College of Fisheries, Guangdong Ocean University, Zhanjiang 524088, China; 2 Guangdong Provincial Key Laboratory of Aquatic Animal Disease Control and Healthy Culture, Zhanjiang 524088, China; 3 Agro-Tech Extension Center of Guangdong Province, Guangzhou 510520, China

## Figure Replacement

In the original publication [1], Figure 1 did not adequately preserve the fine histological detail present in the source images, and the two 50 µm scale bars were incorrect because the image panels were resized during figure assembly without a corresponding adjustment of the scale bar graphics. Figure 1 has therefore been replaced with a composite prepared directly from the original microscopy images without denoising or smoothing, with correctly calibrated scale bars in each panel. The corrected Figure 1 appears below.

## Missing Supplementary Materials

The Supplementary Materials section was not included in the original publication [1], and it should appear as below:

**Supplementary Materials:** The following supporting information can be downloaded at: https://www.mdpi.com/article/10.3390/ani15040515/s1. Table S1: Individual intestinal villus height measurements and fish-level means in the control and *B. coagulans* groups; Table S2: Statistical analysis output for fish-level villus height comparison (IBM SPSS Statistics 24).

The Supplementary Materials have been cited accordingly in Section 3.2 Intestinal Histology, as follows: Histological examination of the intestinal villi revealed that the villus height in the *B. coagulans* group was significantly greater, and the villi appeared more uniform compared to those in the control group (Tables S1 and S2; Figure 1); and Section 4. Discussion as follows: In this study, the intestinal villi of *E. tetradactylum* in the experimental group were taller and more uniform compared to those in the control group (Tables S1 and S2).

The authors state that the scientific conclusions are unaffected. This correction was approved by the Academic Editor. The original publication has also been updated.

## Figures and Tables

**Figure 1:**
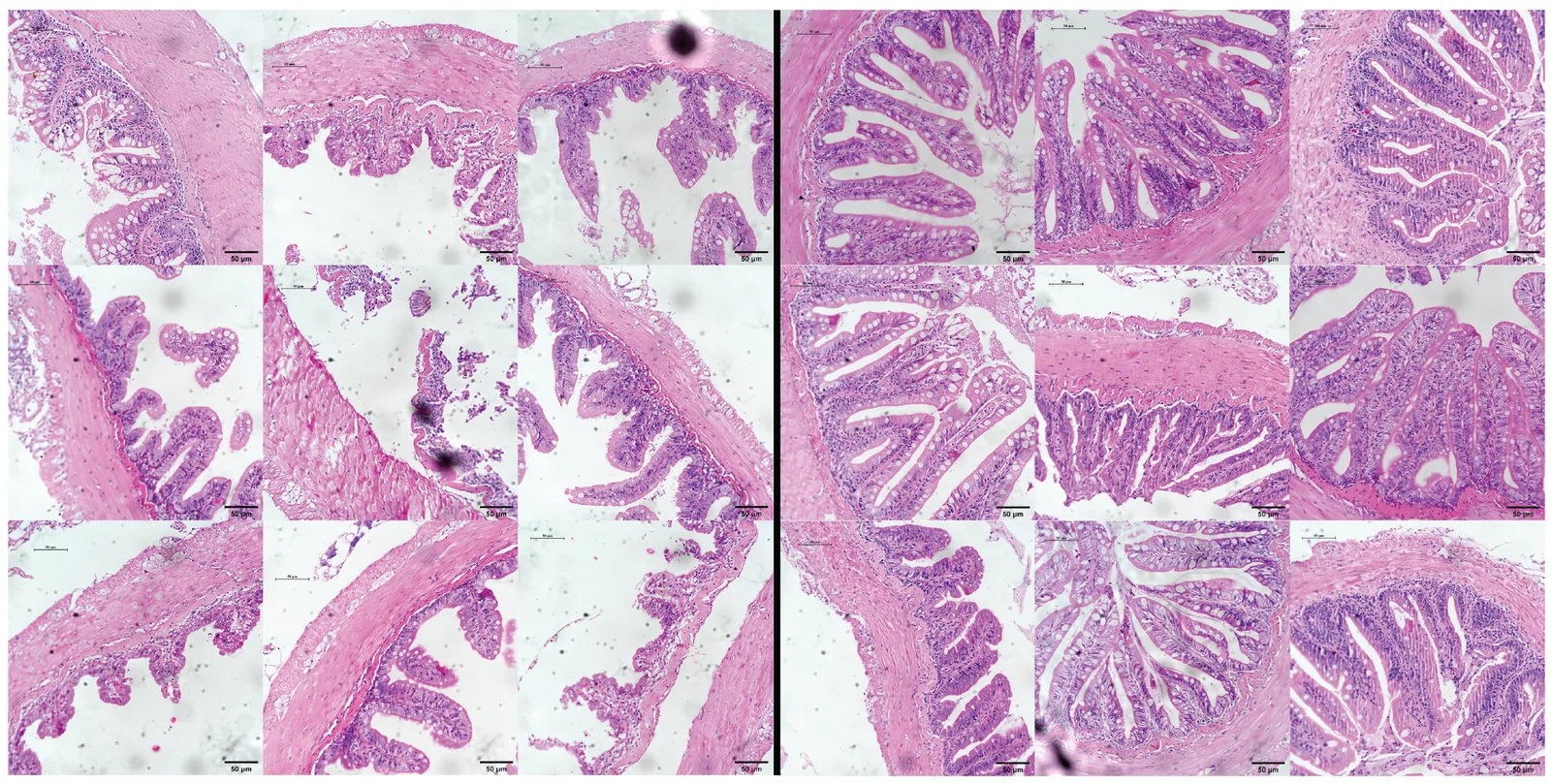
Intestinal morphology of *E. tetradactylum* fed diets supplemented with *B. coagulans*. The left images represent the control group, and the right images represent the experimental group. Scale bars represent 50 μm.
