# Supplementary material for: Effects of Bacillus coagulans on Growth Performance, Digestive Enzyme Activity, and Intestinal Microbiota of the Juvenile Fourfinger Threadfin (Eleutheronema tetradactylum)
Source: Animals (Basel). 2025 Feb 11;15(4):515. doi: 10.3390/ani15040515 (PMC11851985; doi:10.3390/ani15040515)
Supplement: Supplementary file 1 [file animals-15-00515-s001.zip › Table S1.pdf]

Table S1. Individual intestinal villus height measurements and fish-level means in the control and *B. coagulans* groups.

Group	Tank	Fish/Sample ID	Measurement 1 (μm) (1st intact intestinal villus)	Measurement 2 (μm) (2nd intact intestinal villus)	Measurement 3 (μm) (3rd intact intestinal villus)	Fish mean (μm) (average of 3 villi)
Control	C1	C1-F1	171.776	121.747	160.598	151.374
Control	C1	C1-F2	125.492	69.715	106.204	100.470
Control	C1	C1-F3	206.354	175.855	171.805	184.671
Control	C2	C2-F1	115.584	154.668	126.498	132.250
Control	C2	C2-F2	NA	NA	NA	NA
Control	C2	C2-F3	141.104	124.062	128.934	131.367
Control	C3	C3-F1	104.014	106.754	83.391	98.053
Control	C3	C3-F2	123.328	170.097	140.986	144.804
Control	C3	C3-F3	110.977	77.26	49.29	79.176
B. coagulans	B1	B1-F1	249.248	230.42	243.636	241.101
B. coagulans	B1	B1-F2	259.578	265.728	264.809	263.372
B. coagulans	B1	B1-F3	220.342	273.793	207.412	233.849
B. coagulans	B2	B2-F1	257.708	267.366	313.851	279.642
B. coagulans	B2	B2-F2	157.96	147.772	160.809	155.514
B. coagulans	B2	B2-F3	307.215	287.454	232.207	275.625
B. coagulans	B3	B3-F1	124.047	128.024	139.565	130.545
B. coagulans	B3	B3-F2	227.058	216.369	197.437	213.621
B. coagulans	B3	B3-F3	216.415	246.472	192.24	218.376

Note: Villus height was measured using ImageJ and is expressed in μm. For each fish included in the quantitative analysis, three intact intestinal villi were measured, and their mean was calculated as the fish-level value. Sample identifiers indicate the treatment group (C, control; B, *B. coagulans*), tank number, and fish number. For control fish C2-F2, villus boundaries could not be reliably identified; therefore, all three measurements and the corresponding fish-level mean are marked as NA (missing), and no values were imputed. The final dataset comprises 24 villus-height measurements from eight control fish and 27 measurements from nine fish in the *B. coagulans* group.
