# Supplementary material for: Effects of Bacillus coagulans on Growth Performance, Digestive Enzyme Activity, and Intestinal Microbiota of the Juvenile Fourfinger Threadfin (Eleutheronema tetradactylum)
Source: Animals (Basel). 2025 Feb 11;15(4):515. doi: 10.3390/ani15040515 (PMC11851985; doi:10.3390/ani15040515)
Supplement: Supplementary file 1 [file animals-15-00515-s001.zip › Table S2.pdf]

Table S2. Statistical analysis output for fish-level villus height comparison (IBM SPSS Statistics 24).

T-Test

Group Statistics

Group	N	Mean	Std. Deviation	Std. Error Mean
1	8	127.770625	34.0331886	12.0325492
2	9	223.516111	51.5535996	17.1845332

Independent Samples Test

Group	Levene's Test (F)	Levene's Test (Sig.)	t	df	Sig. (2-tailed)	Mean Difference	Std. Error Difference	95% CI Lower	95% CI Upper
Equal variances assumed	1.024	.328	-4.453	15	<.001	-95.7454861	21.5012900	-141.5744008	-49.9165714
Equal variances not assumed			-4.564	13.938	<.001	-95.7454861	20.9783322	-140.7582043	-50.7327679

Independent Samples Effect Sizes

Group	Standardizer [a]	Point Estimate	95% CI Lower	95% CI Upper
Cohen's d	44.2492897	-2.164	-3.365	-.919
Hedges' correction	46.6269083	-2.053	-3.193	-.872
Glass's delta	51.5535996	-1.857	-3.134	-.522

a. The denominator used in estimating the effect sizes.

Cohen's d uses the pooled standard deviation.

Hedges' correction uses the pooled standard deviation, plus a correction factor.

Glass's delta uses the sample standard deviation of the control group.

Note: Group 1 represents the control group, and Group 2 represents the *B. coagulans* group. The variable "Mean" denotes fish-level mean intestinal villus height in µm. Each fish-level value is the average of three villus measurements. The analysis includes eight control fish and nine *B. coagulans*-treated fish. The equal-variances-assumed row gives $t\ (15) = -4.453$, with a two-sided p value of approximately 0.000465.
